# Older age and sex differences in the proportion of vital signs flagged as abnormal

**DOI:** 10.1371/journal.pone.0349936

**Published:** 2026-05-29

**Authors:** Jean-Philippe Emond, Vincent Weng-Jy Cheung, Ariel Mundo Ortiz, Robert Goulden, Sahar Saeed, Philippe Desmarais, Michaël Chassé, Christina Wolfson, Quoc Dinh Nguyen

**Affiliations:** 1 Centre de recherche du Centre hospitalier de l’Université de Montréal, Montreal, Quebec, Canada; 2 Department of Medicine, Division of Geriatrics, Université Laval, Quebec, Quebec, Canada; 3 McGill University Health Centre, McGill University, Montreal, Quebec, Canada; 4 Queen’s University, Public Health Sciences, Kingston, Ontario, Canada; 5 Centre hospitalier de l’Université de Montréal and the Department of Medicine, Faculty of Medicine, School of Public Health, Université de Montréal, Montreal, Quebec, Canada; 6 Research Institute of the McGill University Health Centre and Department of Epidemiology, Biostatistics and Occupational Health, McGill University, Montreal, Quebec, Canada; 7 Division of Geriatrics, Centre hospitalier de l’Université de Montréal, Montreal, Quebec, Canada; 8 Department of Medicine, Université de Montréal, Montreal, Quebec, Canada; University of British Columbia, CANADA

## Abstract

**Background:**

The variability of vital signs thresholds has not been well described in relation to older age and sex assigned at birth. Significant variations may influence clinical interpretation of vital signs, most notably the thresholds at which investigations or treatments are initiated. Our objective was to compare, in stable health status, the percentage of abnormal values (flagging percentage) of standard vital signs thresholds of adults aged 45 years and older and to determine centile-based thresholds by age and sex.

**Methods:**

A retrospective observational study was conducted using data from the Centre for the Integration and Analysis of Medical Data (CITADEL) at the Centre hospitalier de l’Université de Montréal (CHUM), a tertiary care hospital. All inpatients and outpatients, from January 2012 to December 2021, were included. For individuals with multiple visits, a single hospitalization or a single outpatient visit was selected at random for inclusion. To reflect a stable health status, the last vital signs recorded before hospital discharge were selected and in-hospital deaths were excluded. Flagging percentages were identified using a quantile-based approach. Age and sex specific centile-based thresholds were determined by identifying values at the 2.5^th^ and 97.5^th^ centiles. Flagging percentages and centile values for systolic and diastolic blood pressure, heart rate, and temperature were analyzed by age group (45–54, 55–64, 65–74, 75–84, and 85 + years) and sex.

**Results:**

The sample comprised 123,420 individuals, including 90,813 inpatients. Flagging percentages for the standard threshold of upper systolic blood pressure (140 mmHg) ranged from 13.3 (12.7–13.9) % (45-54y assigned female at birth (AFAB)) to 39.8 (38.6–41.0) % (85y+ AFAB). Flagging percentages for standard thresholds ranged between 0.1 (0.1–0.2) to 1.2 (1.0–1.4) % for upper temperature (37.8°C), lower temperature (35.0°C), and lower systolic blood pressure (90 mmHg). Across subgroups, the 97.5^th^ centile-based adapted thresholds varied between 158 (156−159) – 178 (177−180) mmHg for upper systolic blood pressure and between 37.2 (37.1–37.2) – 37.3 (37.3–37.3) °C for upper temperature.

**Conclusions:**

Important differences in vital signs flagging percentages may affect their comparability by vital sign, age, and sex. Standard thresholds may need to be reassessed using clinical outcomes to factor age and sex differences.

## Introduction

Vital signs (VS) are at the centre of patient evaluation and care. “Normal” reference ranges and thresholds were established decades ago using cohorts that may not be representative of older individuals [1], those with multi-comorbidities [2], and more broadly contemporary populations [3]. Both age and sex are associated with physiologic changes that may affect vital sign values and, therefore, the reference ranges and thresholds. Arterial stiffening increases systolic blood pressure (SBP) progressively with age, lowers diastolic blood pressure (DBP), and increases the risk of end-organ damage, particularly for the heart, brain, and kidneys [4]. Aging is also associated with higher resting heart rate (HR), higher blood pressure (BP) variability [5], lower HR variability [6], and lower body temperature [7]. Sex differences in VS have been described, with individuals assigned female at birth (AFAB) having higher HR and temperature, but lower blood pressure than individuals assigned male at birth (AMAB) of the same age [8]. Research has mostly focused on evaluating age and sex differences in the mean values of VS, while differences in abnormal values (values outside the normal reference range) have not been explored. Since extreme values determine reference ranges and thresholds, a better understanding of how they vary by age and sex may improve clinicians’ ability to interpret abnormal vital signs [9].

The flagging percentage of a vital sign is defined as the percentage of patients in a group with vital sign values flagged as abnormal. Thresholds determining flagging percentages must have high sensitivity to detect the presence of an adverse outcome and thus the risk of clinical deterioration, while remaining sufficiently specific [10]. Indeed, low specificity with higher flagging percentages carry risks of information and alert overload for clinicians [11]. Since older age was found to be associated with increased variation in health domains such as physical performance and vital signs [12], adapting vital signs thresholds by age may be more appropriate for older adults than standard thresholds that apply to all adults. Age, and possibly sex- adapted thresholds, may present the opportunity to better inform clinicians of patient status and guide their interventions.

Rather than using standard VS thresholds that apply to all, centile-based approaches have been used [13–15] to predict in-hospital mortality by age group [16] and to develop early warning scores in adult [17]. A centile-based approach would flag a predetermined and constant proportion of VS values. For example, the flagging percentage when abnormal values are beyond the 2.5^th^ and 97.5^th^ centiles is 2.5% for lower values and 2.5% for higher values. Substantial variation in flagging percentages among VS could trigger a reconsideration of standard thresholds.

In this study, we sought to determine whether potential vital signs thresholds in a stable health status differ from standard vital signs thresholds. Our first objective was to compare the flagging percentage of standard thresholds of VS according to age and sex in a contemporary adult population of 45 years of age and older. Our second objective was to determine potential adapted thresholds, specific to age and sex subgroups, based on vital sign centiles.

## Methods

### Study design, population, and data sources

We conducted a retrospective observational study using data from the Centre for the Integration and Analysis of Medical Data (CITADEL), comprising all inpatients and outpatients receiving healthcare services at the Centre hospitalier de l’Université de Montréal (CHUM), a tertiary care hospital. We included patients with vital sign measurements (a set of VS being a specific moment at which at least one of SBP, DBP, HR, or temperature was measured) between January 1^st^, 2012, and December 30^th^, 2021, aged 45 years or older, with data available for date of measurement and sex. There was no reason to exclude partial VS sets, which could include as few as one of the VS, from the analysis. The 45 years inclusion cut-off was chosen to enable comparison between various older adults age groups with middle-aged adults, as in prior studies on aging [18].

The inclusion and exclusion criteria were designed to select a study population reflecting patients who were in the most stable clinical state, with one set of vital signs per patient to prevent overinclusion of values from individuals with multiple medical encounters. Hence, vital signs measurements of patients who visited the emergency department, but were not subsequently admitted, and patients hospitalized in the intensive, or palliative care unit, were excluded. To limit the overinclusion of abnormal VS values, vital signs directly related to the two following contexts of care were removed from the analyses: cardiovascular VS (SBP, DBP, and HR) from cardiac wards (cardiology, cardiac surgery, catheterization laboratory, cardiovascular and thoracic surgery) and temperature from infectious disease wards. For inpatients, the last set of VS prior to discharge, or hospital transfer, was included to reflect clinical stability as proposed previously [9]. For patients with more than one VS set (hospitalization, and/or outpatient visit), a single set was selected at random from all included vital signs measurements using the slice function in R. No identifiable patient information was available in the analysis phase. We followed the STrengthening the Reporting of OBservational studies in Epidemiology (STROBE) statement [19]. This study was conducted in accordance with the Declaration of Helsinki and approved by the CHUM Research Ethics Board (MP-02-2023-10796). Informed consent was waived due to study’s minimal risk and retrospective design.

### Variables and measures

The vital signs of interest were SBP and DBP (in mmHg), HR (in beats per minute [BPM]), and temperature (in degrees Celsius), measured by standard of care methods in a tertiary setting. The hypertension definition of ≥140/90 mmHg remains widespread [20] and is the current gold standard for elevated inpatient blood pressure [21]. The standard vital signs’ values for the lower and upper thresholds were 90 and 140 mmHg for SBP, 60 and 90 mmHg for DBP [20, 22, 23], 60 and 100 BPM for HR [24, 25], and 35.0°C [26, 27] and 37.8°C [28] for oral temperature. Implausible VS values were excluded (i.e., HR < 15 or > 240 BPM, temperature < 25 or > 42°C, SBP > 260 mmHg, and if the DBP was higher than the SBP). Rectal temperatures were converted to oral equivalents by subtracting 0.5°C [29]. Cutaneous and axillary temperatures were excluded since they are generally unreliable [30], and central temperatures were excluded since they are usually measured in critically-ill patients. Age groups were defined using 10-year age strata from age 45 years to 85 years, which were used in prior studies on the impact of age-related outcomes [31].

### Statistical analysis

For the first objective, we used a nonparametric centile-based approach to identify and compare the flagging percentages for standard VS thresholds by age group (45-54y, 55-64y, 65-74y, 75-84y, and 85y+) and sex assigned at birth (female or male). The lower flagging percentage is the percentage of patients with a value below the standard lower threshold and the upper flagging percentage is the percentage of patients with a value above the standard upper threshold. Subgroup analyses for flagging percentages were conducted by setting (inpatient or outpatient) (S Methods Section 1.1). For the second objective, the same nonparametric centile-based approach was used to determine the VS values of the 1^st^, 2.5^th^, 5^th^, 95^th^, 97.5^th^, and 99^th^ centiles, as done in previous studies [9]. The resulting VS values of different centiles corresponded to the centile-based thresholds in our study population. Sensitivity analyses for centile-based adapted thresholds were conducted using two additional approaches: 1) the semiparametric Generalized Additive Models for Location, Scale, and Shape (GAMLSS) [32] model with a Box Cox Power Exponential function and 2) the Hoffman method that assumes a Gaussian distribution and uses central values of a distribution to estimate extreme values [33]. 95% confidence intervals were calculated with Wilson intervals or by bootstrapping. Analyses were conducted using R version 4.3.2 and the specific gamlss package version 5.4–20 (R-Project, cran.r-project.org).

## Results

### Patients and descriptive data

**Fig 1:**
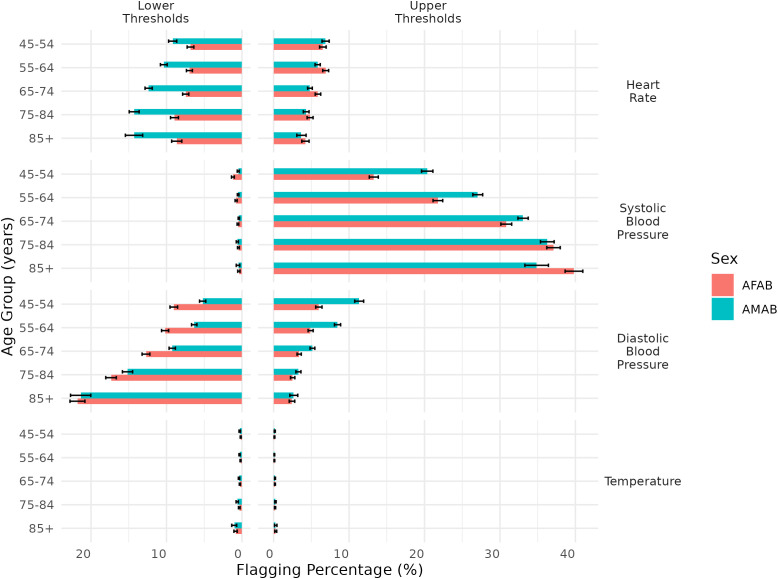
Flagging percentages of standard VS thresholds according to age and sex group. Legend: AFAB: assigned female at birth; AMAB: assigned male at birth; DBP: diastolic blood pressure; HR: heart rate; SBP: systolic blood pressure. Standard vital signs lower and upper thresholds are 90-140 mmHg (SBP), 60-90 mmHg (DBP), 60-100 BPM (HR), and 35.0-37.8°C oral equivalent (temperature). 95% Wilson confidence intervals were calculated.

The study sample included vital signs sets from 123,420 unique individuals following the application of the inclusion and exclusion criteria (S Results Section 2.1). The flowchart showing the building of the cohort based on the CITADEL database is presented in S Fig 1. Descriptive statistics for the study sample are presented in Table 1. Patients were aged between 45 and 108 years with a median and interquartile range (IQR) age of 66 (57, 75) years; 62,089 (50.3%) were AFAB; 90,813 (73.6%) were inpatients; and 32,607 (26.4%) outpatients. The vital sign sets that were taken in cardiac context of care were set as missing from cardiovascular VS analysis for 7,931 patients (6.4%). Vital sign sets that were taken in the infectious disease context of care were set as missing from temperature analysis for 187 patients (0.2%). The 85 years and over group represented 10% of inpatients and 5% of outpatients. The VS median (IQR) values of the full study population and in subgroups according to the setting are presented in Table 1.

**Table 1:** Baseline descriptive characteristics for the study cohort.

Characteristics	All N = 123,420	Inpatients N = 90,813	Outpatients N = 32,607
**Sex assigned at birth n (%)**			
AFAB	62,089 (50.3)	45,035 (49.6)	17,054 (52.3)
AMAB	61,331 (49.7)	45,778 (50.4)	15,553 (47.7)
**Age (IQR)**			
Median in years	66 (57, 75)	66 (57, 76)	64 (56, 73)
**Age Group n (%)**			
45-54 years	23,361 (18.9)	16,425 (18.1)	6,936 (21.3)
55-64 years	33,615 (27.2)	24,048 (26.5)	9,567 (29.3)
65-74 years	34,123 (27.6)	24,842 (27.4)	9,281 (28.5)
75-84 years	22,052 (17.9)	16,855 (18.6)	5,197 (15.9)
85 + years	10,269 (8.3)	8,643 (9.5)	1,626 (5.0)
**Context of care n (%)**			
Cardiac specific	7,931 (6.4)	7,788 (8.6)	143 (0.4)
Infectious disease specific	187 (0.2)	47 (0.1)	140 (0.4)
Other	115,302 (93.4)	82,978 (91.3)	32,324 (99.1)
**Department n (%)**			
Medicine	46,602 (37.8)	37,030 (40.8)	9,572 (29.4)
Surgery	70,106 (56.8)	49,112 (54.1)	20,994 (64.4)
Other	6,712 (5.4)	4,671 (5.1)	2,041 (6.3)
**Vital Sign median (IQR)**			
Heart Rate in BPM N = 111,371	77 (68, 87)	78 (69, 88)	74 (66, 83)
Systolic Blood Pressure in mmHg N = 112,162	129 (116, 143)	127 (115, 142)	132 (120, 146)
Diastolic Blood Pressure in mmHg N = 112,162	73 (66, 80)	72 (65, 79)	76 (69, 83)
Temperature in °C N = 103,395	36.6 (36.4, 36.8)	36.6 (36.4, 36.8)	36.6 (36.3, 36.8)

Legend: AFAB: assigned female at birth; AMAB: assigned male at birth; BPM: Beats per minute; IQR: Interquartile range; Cardiac context of care comprises cardiology, catheterization laboratory, cardiac surgery, and cardiothoracic surgery; Infectious disease context of care comprises infectious disease only; Other contexts of care comprise all contexts other than cardiac and infectious disease; Visits in the departments of radiology, psychiatry, radio-oncology, and nursing were included in “Other”. The number of vital signs values vary, as some vital signs sets were partial (comprising one, two, or three vital signs), and some were set as missing (temperature in infectious disease context of care; SBP, DBP, and HR in cardiac context of care).

### Flagging percentages of standard thresholds

The proportion of participants in each subgroup (by threshold, vital sign, age, and sex) whose measurements exceeded or fell below standard clinical cut-points is summarized in S1 Table. For heart rate, 3.6 (3.1–4.3) – 6.9 (6.6–7.4) % exceeded the upper threshold of 100 beats per minute, whereas 6.8 (6.4–7.3) – 14.3 (13.6–14.9) % were below the lower threshold of 60 BPM. For systolic blood pressure, 13.3 (12.7–13.9) –39.8 (38.6–41.0) % exceeded 140 mmHg, while 0.4 (0.3–0.6) – 1.2 (1.0–1.4) % were below 90 mmHg. Similarly, 2.4 (2.1–2.8) – 11.3 (10.7–11.9) % of participants exceeded the diastolic blood pressure threshold of 90 mmHg, and 5.2 (4.8–5.6) – 21.8 (20.8–22.8) % fell below 60 mmHg. Finally, 0.1 (0.1–0.2) – 0.3 (0.2–0.4) % registered temperatures above the upper threshold of 37.8°C, and 0.1 (0.1–0.2) – 1.0 (0.7–1.4) % were below 35.0°C.

Fig 1 illustrates the differences in flagging percentages for each standard threshold, stratified by vital sign, age group, and sex. Low heart rate was flagged more frequently in older adults, reaching 14.3 (13.6–14.9) % in AMAB aged 85 years and above. High systolic blood pressure also increased with age, particularly among AFAB, from 13.3 (12.7–13.9) % in those aged 45–54 to 39.8 (38.6–41.0) % in those aged 85 and older. Low diastolic blood pressure flags were higher in the oldest age groups, rising to 21.8 (20.8–22.8) % in AFAB and 21.3 (20.0–22.7) % in AMAB aged 85 and above. By contrast, flagging percentages for low SBP, as well as high and low HR and temperature, were similar across all age and sex groups. Overall flagging percentages ranged from 0.1 (0.1–0.2) % (upper temperature in AMAB and AFAB aged 45–64) to 39.8 (38.6–41.0) % (high SBP in AFAB aged 85+). In older AFAB (85+), high SBP (39.8%) was flagged roughly 100 times more often than low SBP (0.4%) or high temperature (0.3%). Notably, high SBP flags were most common, whereas low SBP and both high and low temperature flags were the least common across all VS. AMAB were found to have a higher flagging percentage of high SBP in younger groups (<75y), which shifted at age 75y, with AFAB being those with a higher flagging percentage of high SBP in the 85 + y group. AMAB consistently displayed lower heart rate than AFAB.

### Subgroup analysis of flagging percentages of standard thresholds by setting

**Fig 2:**
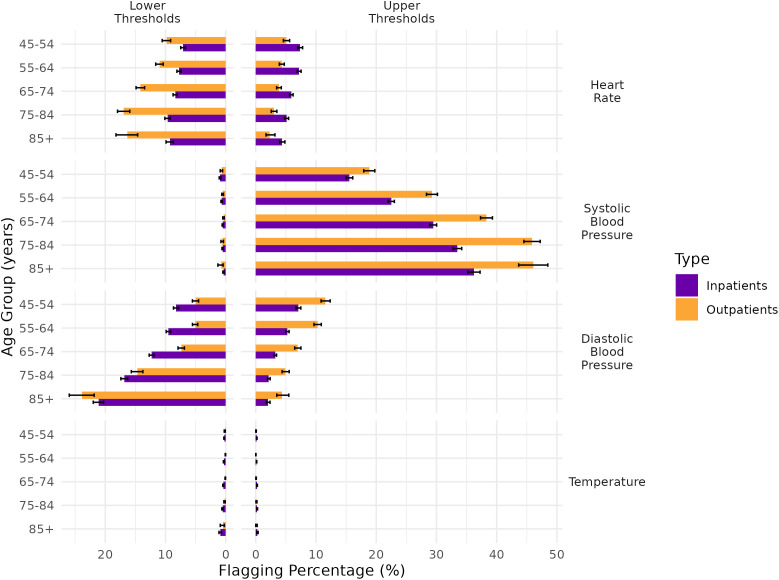
Flagging percentages of lower and upper standard thresholds by setting and according to age group. Legend: DBP: diastolic blood pressure; HR: heart rate; SBP: systolic blood pressure. Standard vital signs lower and upper thresholds are 90-140 mmHg (SBP), 60-90 mmHg (DBP), 60-100 BPM (HR), and 35.0-37.8°C oral equivalent (temperature). 95% Wilson confidence intervals were calculated.

Flagging percentages for heart rate were higher, and those for blood pressure were lower, among inpatients compared to outpatients; however, overall trends were similar across both settings (Fig 2). In inpatients aged 65 years or older, 30% and more met the upper systolic blood pressure threshold, which rose to nearly 40% in those aged 85 years and above. Among outpatients aged 65 years or older, 40% and more exceeded the upper SBP threshold, approaching 50% in those aged 85 years and above.

### Centile-based adapted thresholds specific for age and sex

**Fig 3:**
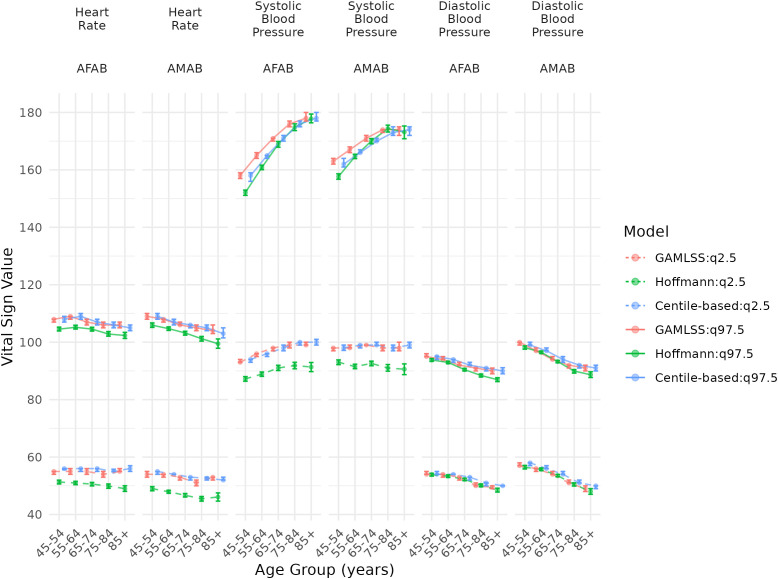
Adapted thresholds of blood pressure and heart rate based on the 2.5^th^ and 97.5^th^ centiles determined by different statistical method and according to age group and sex. Legend: AFAB: assigned female at birth; AMAB: assigned male at birth; DBP: diastolic blood pressure; GAMLSS: Generalized Additive Models for Location, Scale, and Shape; HR: heart rate; SBP: systolic blood pressure. The units for SBP and DBP are mmHg and for HR are beats per minute. 95% Confidence intervals were bootstrapped with 1000 replicates.

**Fig 4:**
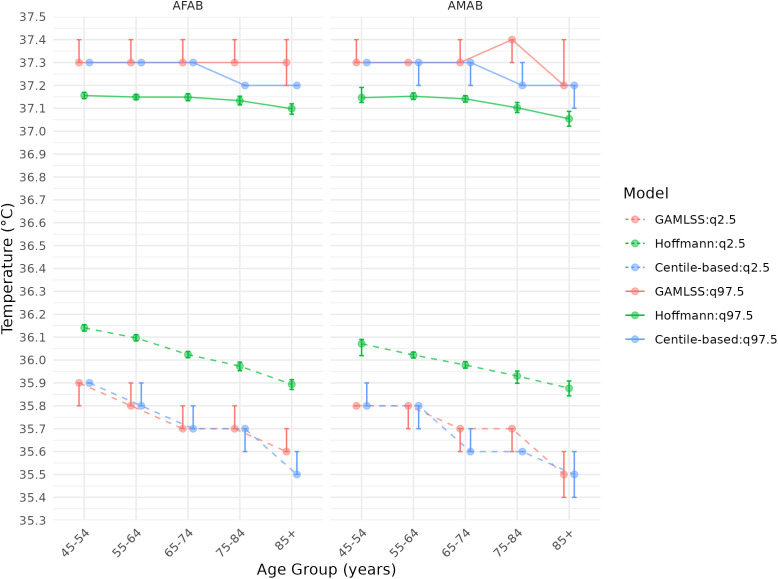
Adapted thresholds of temperature based on the 2.5^th^ and 97.5^th^ centiles determined by different statistical method and according to age group and sex. Legend: AFAB: assigned female at birth; AMAB: assigned male at birth; GAMLSS: Generalized Additive Models for Location, Scale, and Shape. 95% Confidence intervals were bootstrapped with 1000 replicates.

Figs 3 and 4 present the 97.5^th^ and 2.5^th^ centiles thresholds for each vital sign according to three statistical methods. The sensitivity analysis using GAMLSS and the Hoffman approach closely mirrored the centile-based findings (Figs 3 and 4). Tables 2 and 3 provide detailed VS values for these centile-based thresholds. Notably, the upper adapted systolic blood pressure threshold increased with age, ranging from 158 (156−159) to 178 (177−180) mmHg in AFAB and 162 (161−164) to 174 (172−175) mmHg in AMAB, while the lower threshold rose from 94 (93−94) to 100 (99−101) mmHg in AFAB and 98 (97−99) to 99 (98−100) mmHg in AMAB. The upper adapted temperature thresholds across all subgroups ranged from 37.2 (37.1–37.2) to 37.3 (37.3–37.3) °C, and the lower thresholds from 35.5 (35.4–35.6) to 35.9 (35.9–35.9) °C.

**Table 2:** Adapted thresholds based on corresponding blood pressure values of centiles of the study cohort.

		AFAB (age group in years)					AMAB (age group in years)				
**VS**	**C**	**45-54**	**55-64**	**65-74**	**75-84**	**85+**	**45-54**	**55-64**	**65-74**	**75-84**	**85+**
SBP (mmHg)	99	167 (165-168)	174 (171-175)	179 (177-180)	184 (183-186)	187 (184-188)	172 (169-173)	175 (173-176)	179 (177-180)	181 (180-182)	179 (177-182)
	97.5	158 (156-159)	165 (164-165)	171 (170-172)	176 (175-177)	178 (177-180)	162 (161-164)	166 (166-167)	170 (170-171)	173 (172-175)	174 (172-175)
	95	151 (150-152)	158 (157-158)	164 (164-165)	169 (168-170)	171 (170-172)	156 (155-157)	160 (159-160)	164 (163-165)	167 (166-168)	168 (166-169)
	5	97 (97-98)	100 99-100)	102 (102-103)	104 (103-104)	104 (104-105)	101 (101-102)	102 (102-103)	103 (103-104)	103 (102-104)	103 (102-104)
	2.5	94 (93-94)	96 (95-96)	98 (97-99)	100 (99-100)	100 (99-101)	98 (97-99)	99 (98-99)	100 (99-100)	98 (97-99)	99 (98-100)
	1	90 (90-91)	92 (91-92)	934 (93-94)	95 (94-96)	95 (94-96)	94 (93-94)	94 (93-95)	94 (93-95)	93 (93-94)	94 (92-96)
DBP (mmHg)	99	99 (99-100)	98 (97-99)	97 (96-97)	95 (94-96)	95 (94-96)	105 (104-106)	100 (100-102)	98 (98-99)	96 (95-97)	96 (95-98)
	97.5	95 (94-96)	94 (93-94)	92 92-93)	90 (90-91)	90 (89-91)	99 (99-100)	97 (97−97)	94 (93-95)	92 (91-92)	91 (89-92)
	95	92 (91-92)	90 (90-91)	89 (88-89)	87 (86-87)	86 (85-87)	96 (95-96)	94 (93-94)	91 (90-91)	88 (88-89)	87 (85-88)
	5	58 (57 –58)	57 (57 –58)	56 (55 –56)	54 (53 –54)	52 (52 –53)	60 (60 –61)	60 (60 –60)	58 (57 –58)	55 (54 –55)	53 (52 –53)
	2.5	54 (54 –55)	54 (54 –54)	53 (52 –53)	51 (50 –51)	50 (50 –50)	58 (57 –58)	56 (56 –57)	54 (54 –55)	51 (51 –52)	50 (49 –50)
	1	51 (50 –52)	50 (50 –50)	50 (49 –50)	47 (47 –48)	47 (45 –48)	54 (53 –55)	52 (52 –53)	50 (50 –51)	48 (47 –49)	46 (44 –48)

Legend: AFAB: assigned female at birth; AMAB: assigned male at birth; C: centile; DBP: diastolic blood pressure; SBP: systolic blood pressure; VS: vital sign. Adapted thresholds with a global flagging percentage of 5% would correspond to the values of the 2.5^th^ and 97.5^th^ centiles (rows in grey). 95% Confidence intervals were bootstrapped with 1000 replicates.

**Table 3:** Adapted thresholds based on corresponding heart rate and temperature values of centiles of the study cohort.

		AFAB (age group in years)					AMAB (age group in years)				
VS	C	45-54	55-64	65-74	75-84	85+	45-54	55-64	65-74	75-84	85+
HR (BPM)	99	114 (113-116)	115 (114-116)	113 (112-114)	112 (110-114)	111 (110-112)	115 (114-117)	114 (112-115)	112 (111-112)	111 110-112)	112 (109-115)
	97.5	108 (107-109)	109 (108-110)	107 (106-108)	106 (105-107)	105 (104-106	109 (108-110)	107 (106-108)	106 (105-106)	105 (104-106)	103 (102-105)
	95	103 (102-103)	103 (103-104)	102 (101-103)	100 (100-101)	100 (98-100)	103 (103-104)	102 (101-102)	100 (100-101)	100 (99-100)	98 (97-99)
	5	59 (59 –60)	59 (59 –60)	59 (58 –59)	58 (58 –58)	59 (58 –59)	58 (57– 58)	57 (57 –57)	56 (55 –56)	55 (55 –56)	56 (55 –56)
	2.5	56 (55 –56)	56 (55 –56)	56 (55 –56)	55 (55 –55)	56 (55 –56)	55 (54– 55)	54 (54 –54)	53 (52 –53)	53 (52 –53)	52 (52 –53)
	1	52 (52 –53)	52 (52 –53)	52 (52 –53)	52 (51 –52)	53 (52 –54)	51 (51– 52)	51 (50 –51)	50 (49 –50)	49 (49 –50)	50 (48 –50)
T° (°C)	99	37.4 (37.4-37.5)	37.4 (37.4-37.5)	37.5 (37.4-37.5)	37.4 (37.3-37.4)	37.4 (37.3-37.5)	37.5 (37.5-37.5)	37.4 (37.4-37.5)	37.5 (37.4-37.5)	37.4 (37.4-37.5)	37.4 (37.3-37.4)
	97.5	37.3 (37.3-37.3)	37.3 (37.3-37.3)	37.3 (37.3-37.3)	37.2 (37.2-37.2)	37.2 (37.2-37.2)	37.3 (37.3-37.3)	37.3 (37.2-37.3)	37.3 (37.2-37.3)	37.2 (37.2-37.3)	37.2 (37.1-37.2)
	95	37.2 (37.2-37.2)	37.2 (37.1-37.2)	37.1 (37.1-37.2)	37.1 (37.1-37.1)	37.10 (37.0-37.1)	37.2 (37.1-37.2)	37.1 (37.1-37.1)	37.1 (37.1-37.1)	37.1 (37.1-37.1)	37.0 (37.0-37.1)
	5	36.0 (36.0-36.0)	36.0 (36.0-36.0)	36.0 (36.0-36.0)	36.0 (35.9-36.0)	35.9 (35.8-35.9)	36.0 (36.0-36.0)	36.0 (36.0-36.0)	35.9 (35.9-35.9)	35.9 (35.8-35.9)	35.7 (35.6-35.8)
	2.5	35.9 (35.9-35.9)	35.8 (35.8-35.9)	35.7 (35.7-35.8)	35.7 (35.6-35.7)	35.5 (35.5-35.6)	35.8 (35.8-35.9)	35.8 (35.7-35.8)	35.6 (35.6-35.7)	35.6 (35.6-35.6)	35.5 (35.4-35.6)
	1	35.6 (35.6-35.7)	35.5 (35.5-35.6)	35.5 (35.5-35.5)	35.5 (35.4-35.5)	35.2 (35.0-35.3)	35.6 (35.5-35.6	35.5 (35.5-35.5)	35.4 (35.4-35.5)	35.3 (35.2-35.4)	35.1 (35.0-35.2)

Legend: AFAB: assigned female at birth; AMAB: assigned male at birth; BPM: beats per minute; C: centile; HR: heart rate; T°: temperature; VS: vital sign. Adapted thresholds with a global flagging percentage of 5% would correspond to the values of the 2.5^th^ and 97.5^th^ centiles (rows in grey). 95% Confidence intervals were bootstrapped with 1000 replicates.

### Sensitivity analyses

The hypertension threshold recommended by the ACC/AHA of 130/80 mmHg yielded significantly different results. The results according to age group and sex are displayed in S2 Table and S2 Fig. The flagging percentage, with the 130 mmHg compared with the 140 mmHg upper SBP threshold, in AFAB of 45–54 years was 27.0 (26.2–27.8) % vs 13.3 (12.7–13.9) %. In the 85 + years groups, they were 57.5 (56.3–58.7) % vs 39.8 (38.6–41.0) %. In AMAB, they were in the 45–54 years groups of 38.6 (37.7–39.5%) vs 20.3 (19.6–21.1) %, and in the 85 + years groups of 52.2 (50.5–53.8) % vs 34.8 (33.3–36.4) %. The trends were similar in the DBP groups. The sensitivity analyses according to setting and age groups are showed in S3 Table and S3 Fig. The increase in flagging percentage in the 85 + years groups was 53.8 (52.7–54.8) % in inpatients and of 63.6 (61.2–65.9) % in outpatients. For DBP, the flagging percentages of high DBP were significantly higher in all age groups compared to the standard threshold.

Sensitivity analyses were conducted to determine the flagging percentage and the adapted thresholds with all patients of infectious disease (S4 and S5 Tables and S4 Fig) and of cardiac (S6 and S7 Tables and S5 Fig) contexts of care. The inclusion of patients in infectious disease context of care did not influence both flagging percentage and adapted thresholds for temperature. The inclusion of patients in cardiac context of care did not influence both flagging percentage and adapted thresholds of HR and DBP. For SBP, there was a slight decrease in the flagging percentage of the upper SBP threshold (140 mmHg) in 85 + years AFAB, with a decrease from 39.8 (38.6–41.0) % to 37.8 (36.6–38.9) %.

## Discussion

In this large study based on electronic health records of real-world patients aged 45 years and older, we have shown that age and sex affect the percentage of vital signs flagged as abnormal. In fact, flagging percentages of standard thresholds varied substantially across VS, age, and sex groups, ranging between 0.1% and 39.8%. There were disproportionately high flagging percentages, particularly in older age groups and in AFAB, for the standard upper threshold of SBP compared with all other thresholds. Conversely, flagging percentages for standard thresholds of lower and upper temperature and lower SBP were very low across all subgroups. Hypothetical centile-based adapted thresholds using the 97.5^th^ centiles increased between the youngest and oldest age groups for the upper thresholds for SBP, increasing from 158 to 178 mmHg in AFAB and from 162 to 174 mmHg in AMAB. The oral equivalent temperature centile-based adapted thresholds using the 2.5^th^ and 97.5^th^ centiles differed from standard thresholds at 35.5–35.9°C vs 35.0°C (lower threshold) and 37.2–37.3°C vs 37.8°C (upper threshold).

The variability and difference in magnitude of flagging percentages using standard thresholds suggest that these thresholds might be reconsidered according to age and sex. Although flagging percentages of standard thresholds are high for SBP, it may be adequate. Studies with clinical outcomes and different thresholds will be needed to determine whether standard thresholds should be modified for age and sex-specific adapted thresholds. The current recommended definition for hypertension is > 130/80 mmHg [34], with values ≥120/80 mmHg now considered elevated and abnormal. In our study cohort, with the application of the 130/80 mmHg threshold, the increase in flagging percentage of SBP is of approximately 20% and of DBP of approximately 4 times higher in all age and sex groups. However, when considering inpatients specifically, there is little guidance for the management of asymptomatic high SBP [35]. Indeed, there are currently no specific normal values of blood pressure for the inpatient population [36]. Whereas low and strict targets for SBP provide benefits for outpatients [37, 38], the interpretation and management of SBP and other VS may vary by time and place. As to sex differences, the shift observed in higher upper SBP flagging percentage from AMAB to AFAB may be in part attributable to menopause and hormone changes [39]. While our subgroup analysis seemed to show that the upper SBP flagging percentage is even higher in outpatients, the flagging percentage of inpatients is still close to 40% in the 85 + group. An increase of the standard upper threshold for SBP with age has been previously advocated [40] and is supported by studies showing increased adverse events when treating asymptomatic hypertension in this population [21, 41]. Although it may not be wise to tolerate high SBP values, even in older asymptomatic inpatients, further studies are needed to fill this gap in knowledge and determine optimal thresholds for treatment [42].

With regards to thresholds for hospitalized older adults, the very low flagging percentages for upper temperature, lower temperature, and lower SBP suggest those thresholds may be too low. Previous work has shown that the optimal lower SBP threshold for trauma patients increased with age from 96 mmHg to 117 mmHg in those 65 years and above [43] and low sensitivity in nursing homes for sepsis [44], and for fever due to COVID-19 infection [45]. High specificity of standard temperature thresholds has been previously highlighted [3, 46, 47], with the 99^th^ centile varying between 36.8°C and 37.9°C according to subgroups [3]. Adapted thresholds based on the values of the 2.5^th^ and 97.5^th^ centiles in the hospital setting concurs with evidence showing that odds of in-hospital mortality were lower when SBP was > 140 mmHg and higher when SBP was < 100 mmHg, or when temperature was < 35.5°C in the emergency department [48].

Our finding that VS flagged as abnormal may vary depending on specific VS, older age, and sex have potential implications on clinical practice. The striking variation in flagging percentages, from 0.1 to 39.8%, and the risk of alert fatigue with high flagging percentages [11], may justify the reconsideration and comparative adjustment of vital sign thresholds. For example, the frequent focus on high SBP may cause relevant temperatures or lower SBP, both flagged and unflagged, to be overlooked. Further studies evaluating the association between VS thresholds and clinical outcomes are warranted to determine whether standard VS thresholds could be improved for specific subgroups and whether optimal thresholds would be symmetrical (i.e., different flagging percentages for the upper and lower thresholds) according to centiles.

### Strengths and limitations

The main strengths of our study include the focus on VS flagging percentages variations according to age and sex group. The large population-based CITADEL cohort reflects real-world practice and enhances external validity. Nevertheless, limitations should be acknowledged. First, large databases such as CITADEL carry risk of biases, such as ascertainment bias with data available in a hospital setting (inpatient or outpatient), measurement error due to real-world measurements, and missing data due to both data collection and availability [49]. None of those biases are directly associated with age or sex. Second, although we did not use the BP and HR of patients in cardiac contexts of care, the specific impact of comorbidities and medications outside of cardiac contexts of care were not explicitly considered. The minimal changes observed with the inclusion of patients in cardiac context of care supports generalization of our findings to most patients. The exclusion of patients in the emergency department, intensive care unit, and palliative care unit may also introduce spectrum bias that compresses tails of distribution by reducing the number of extreme values. Findings in this study reflect vital signs distributions biased towards “normal values”. Third, even though we only included the last set of VS before discharge for inpatients, our study population cannot be considered fully “normal.” Higher prevalence of undiagnosed hypertension [50] and undertreatment due to therapeutic inertia for older adults [51] may have contributed to higher SBP values. Selection bias may have skewed the adapted thresholds towards a more comorbid population using a tertiary hospital setting sample. The random selection of a single VS set per patient does not fully address selection biases, as there may not be consistency across all VS sets. Also, the database does not provide information on conditions and procedures of the VS measurements. Further studies aiming at identifying thresholds in specific settings, medical conditions, and with documentation of measurement procedures may address concerns related to information and selection biases. Fourth, VS centiles values in the extremes could be sensitive to the statistical method used, but sensitivity analyses comparing three statistical methods showed similar findings. Finally, replication is warranted to address generalizability issues.

## Conclusion

In this large, real-world study of hospitalized and outpatient adults, we observed substantial variability in how standard vital sign thresholds flag abnormal measurements across different age and sex groups. These findings supports the potential need for adapted VS thresholds, especially for older, comorbid populations, and highlight the importance of validating such adaptations against relevant clinical outcomes. Future research should focus on determining whether these modified thresholds can reduce unnecessary alerts, focusing on the patients that need acute patient care.

## Supporting information

S1 Fig Flowchart showing the building of the cohort. Legend: CITADEL: Centre for the Integration and Analysis of Medical Data; VS: Vital sign.
(PDF)

S2 Fig Flagging percentages of lower and upper standard thresholds by sex for 130/80 mmHg SBP and DBP upper thresholds. Legend: AFAB: assigned female at birth; AMAB: assigned male at birth; DBP: diastolic blood pressure; SBP: systolic blood pressure. Standard vital signs upper thresholds are 90–140 mmHg and 80–130 mmHg (DBP-SBP). 95% Wilson confidence intervals were calculated.
(TIFF)

S3 Fig Flagging percentages of lower and upper standard thresholds by age group and setting for 130/80 mmHg SBP and DBP upper thresholds. Legend: DBP: diastolic blood pressure; SBP: systolic blood pressure. Standard vital signs upper thresholds are 90–140 mmHg and 80–130 mmHg (DBP-SBP). 95% Wilson confidence intervals were calculated.
(TIFF)

S4 Fig Flagging percentages of lower and upper standard thresholds for temperature by sex in main analysis and when including infectious contexts. Legend: AFAB: assigned female at birth; AMAB: assigned male at birth. Standard vital signs lower and upper thresholds are 35.0–37.8°C oral equivalent (temperature). 95% Wilson confidence intervals were calculated.
(TIFF)

S5 Fig Flagging percentages of lower and upper standard thresholds by sex for main analysis and when including cardiac contexts. Legend: AFAB: assigned female at birth; AMAB: assigned male at birth. Standard vital signs lower and upper thresholds are 90–140 mmHg (SBP), 60–90 mmHg (DBP) and 60–100 BPM (HR). 95% Wilson confidence intervals were calculated.
(TIFF)

S1 Table Flagging percentages of standard VS thresholds by age group and sex in our study cohort. Legend: AFAB: assigned female at birth; AMAB: assigned male at birth; BPM: beats per minute; DBP: diastolic blood pressure; F: female; HR: heart rate; M: male; SBP: systolic blood pressure. The columns representing the upper thresholds are in grey. The centile-based approach was used. 95% Wilson confidence intervals were calculated.
(DOCX)

S2 Table Flagging percentages of standard VS thresholds by age group and sex using 130/80 mmHg SBP/DBP upper thresholds. Legend: AFAB: assigned female at birth; AMAB: assigned male at birth; SBP: systolic blood pressure; VS: vital sign. 95% Wilson confidence intervals were calculated.
(DOCX)

S3 Table Flagging percentages of standard VS thresholds by age group and setting for 130/80 mmHg SBP/DBP upper thresholds. Legend: AFAB: assigned female at birth; AMAB: assigned male at birth; SBP: systolic blood pressure; VS: vital sign. 95% Wilson confidence intervals were calculated.
(DOCX)

S4 Table Flagging percentages of standard temperature thresholds by sex when including infectious contexts. Legend: AFAB: assigned female at birth; AMAB: assigned male at birth. 95% Wilson confidence intervals were calculated.
(DOCX)

S5 Table Adapted thresholds based on corresponding VS values of centiles when including infectious contexts. Legend: AFAB: assigned female at birth; AMAB: assigned male at birth; BPM: beats per minute; C: centile; T°: temperature; VS: vital sign. Adapted thresholds with a global flagging percentage of 5% would correspond to the values of the 2.5^th^ and 97.5^th^ centiles (rows in grey). 95% Confidence intervals were bootstrapped with 1000 replicates.
(DOCX)

S6 Table Flagging percentages of standard VS thresholds by sex when including cardiac contexts. Legend: AFAB: assigned female at birth; AMAB: assigned male at birth; BPM: beats per minute; DBP: diastolic blood pressure; HR: heart rate; SBP: systolic blood pressure; VS: vital sign. 95% Wilson confidence intervals were calculated.
(DOCX)

S7 Table Adapted thresholds based on corresponding VS values of centiles when including cardiac contexts. Legend: AFAB: assigned female at birth; AMAB: assigned male at birth; BPM: beats per minute; C: centile; DBP: diastolic blood pressure; HR: heart rate; SBP: systolic blood pressure; VS: vital sign. Adapted thresholds with a global flagging percentage of 5% would correspond to the values of the 2.5^th^ and 97.5^th^ centiles (rows in grey). 95% Confidence intervals were bootstrapped with 1000 replicates.
(DOCX)

S1 File eMethods. (DOCX)

S2 File eResults. (DOCX)
