# Supplementary figures and images for: Older age and sex differences in the proportion of vital signs flagged as abnormal
Source: PLoS One. 2026 May 29;21(5):e0349936. doi: 10.1371/journal.pone.0349936 (PMC13221073; doi:10.1371/journal.pone.0349936)

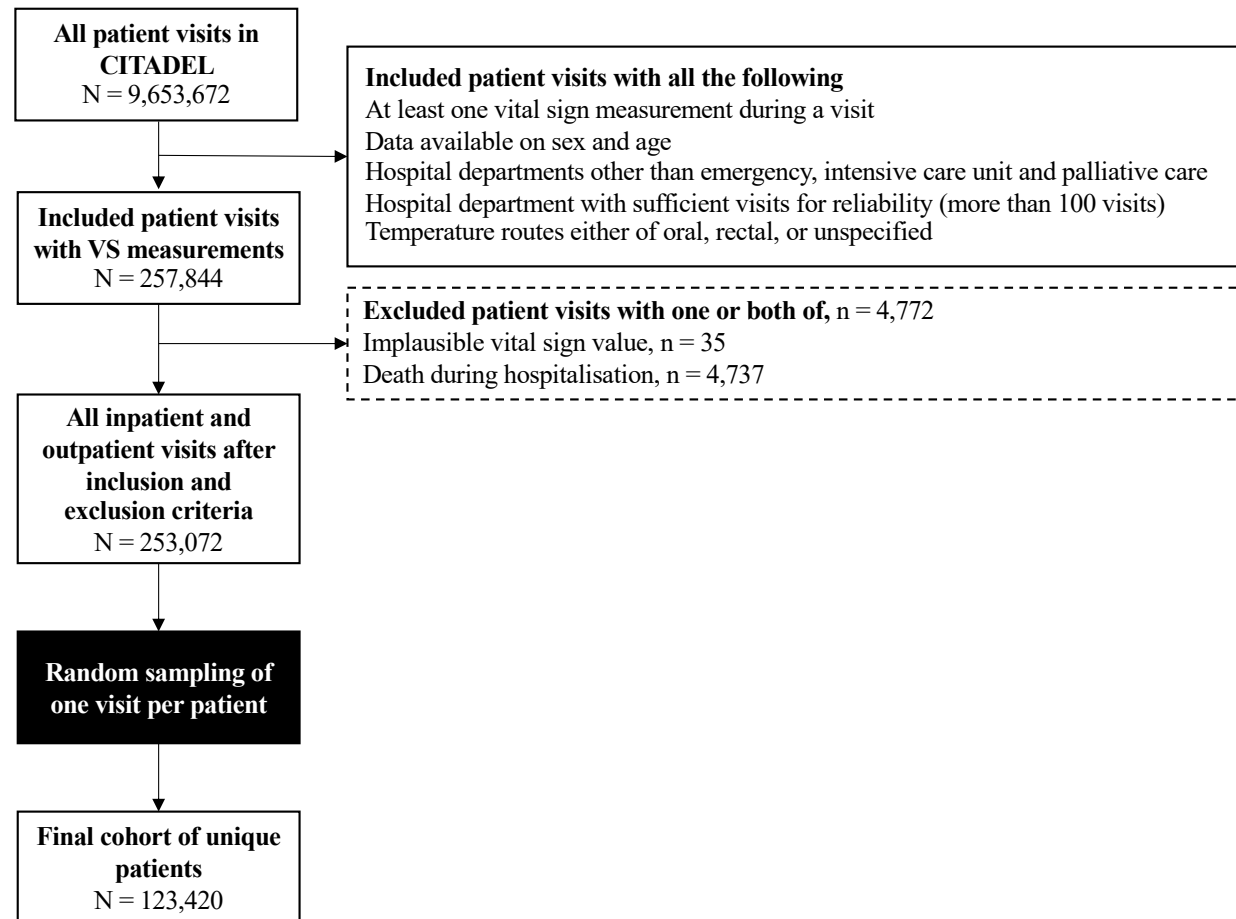

Supplement: S1 Fig — Legend: CITADEL: Centre for the Integration and Analysis of Medical Data; VS: Vital sign. (PDF) [file pone.0349936.s001.pdf]

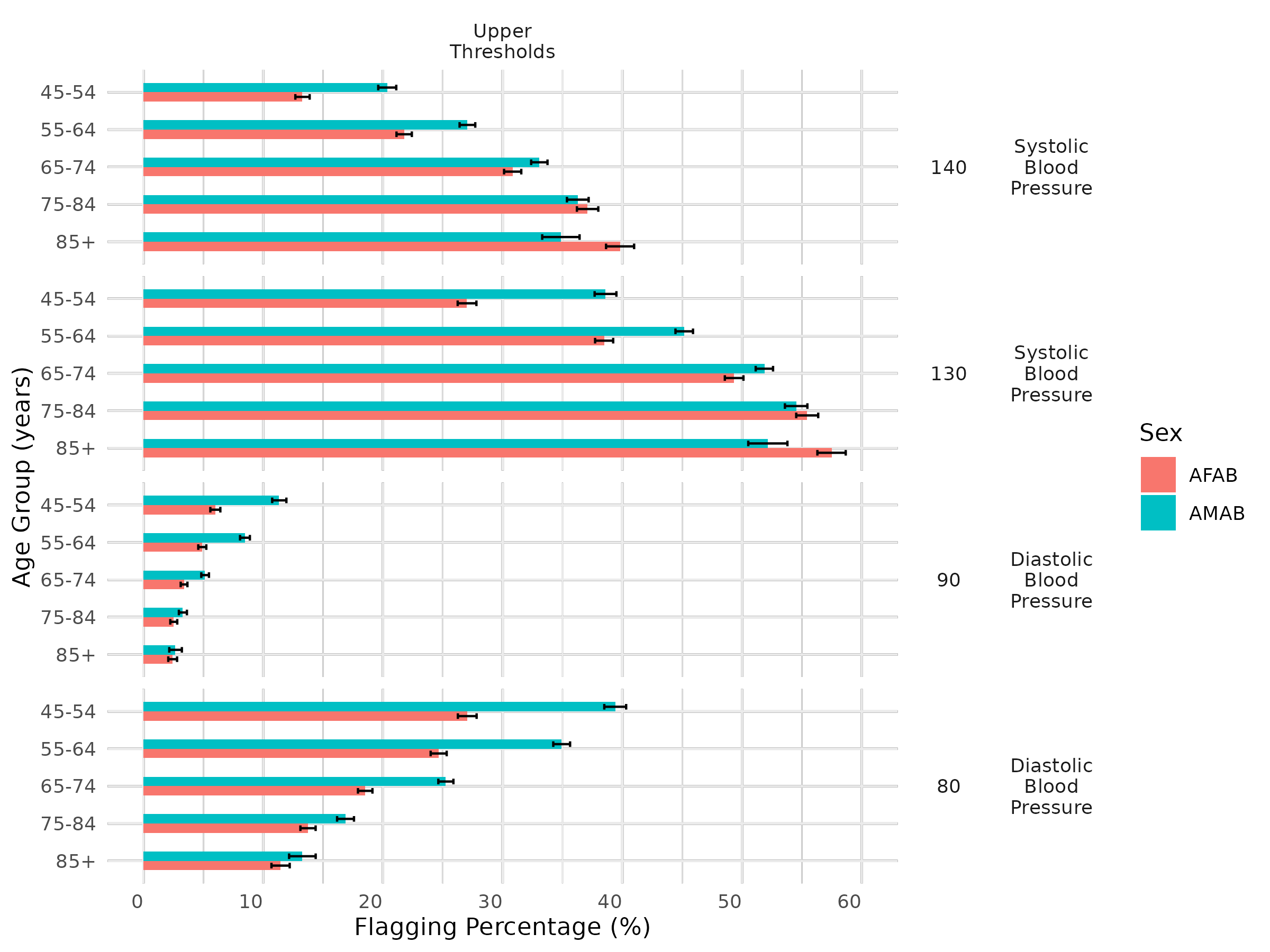

Supplement: S2 Fig — Legend: AFAB: assigned female at birth; AMAB: assigned male at birth; DBP: diastolic blood pressure; SBP: systolic blood pressure. Standard vital signs upper thresholds are 90–140 mmHg and 80–130 mmHg (DBP-SBP). 95% Wilson confidence intervals were calculated. (TIFF) [file pone.0349936.s002.tiff]

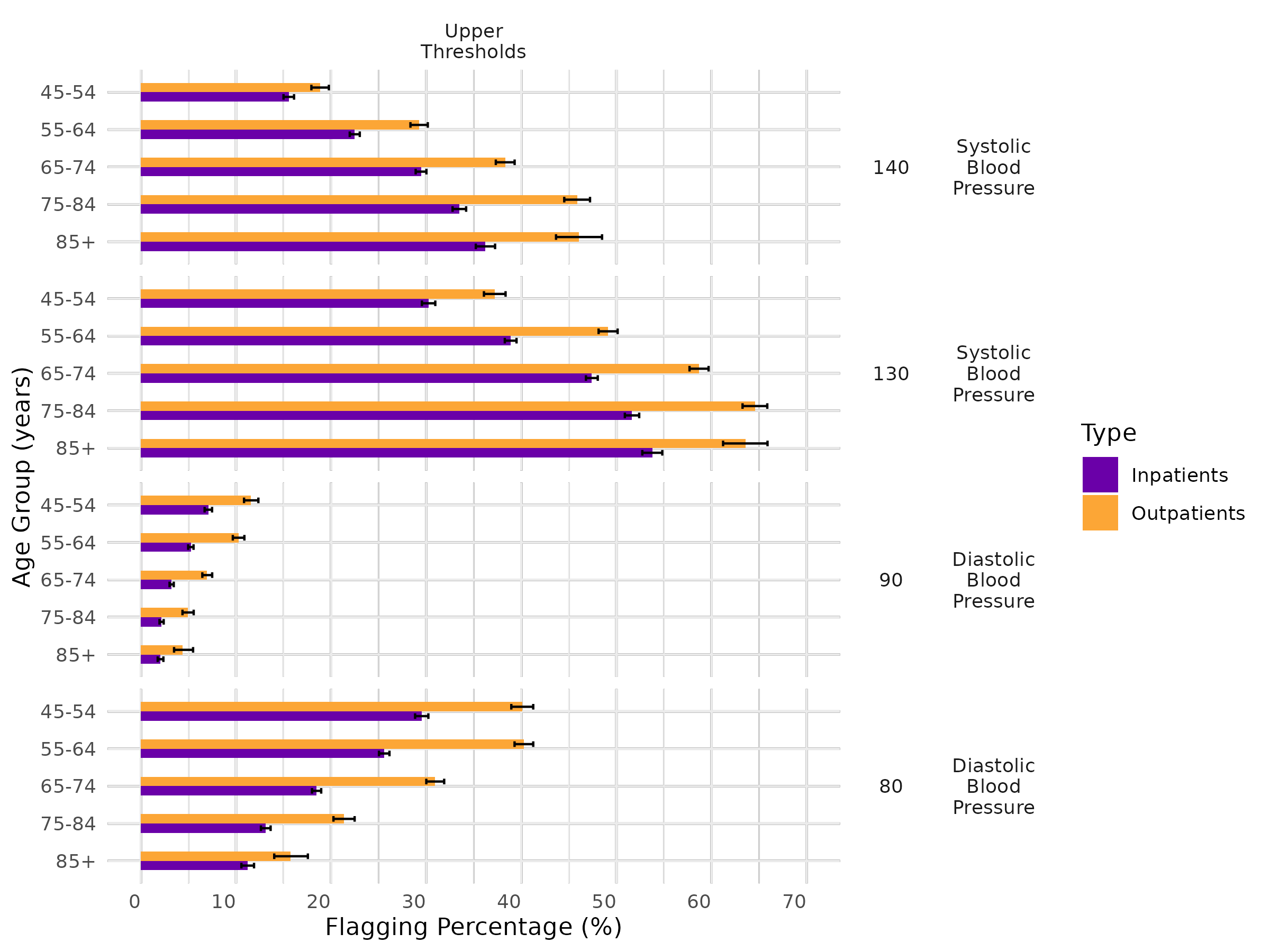

Supplement: S3 Fig — Legend: DBP: diastolic blood pressure; SBP: systolic blood pressure. Standard vital signs upper thresholds are 90–140 mmHg and 80–130 mmHg (DBP-SBP). 95% Wilson confidence intervals were calculated. (TIFF) [file pone.0349936.s003.tiff]

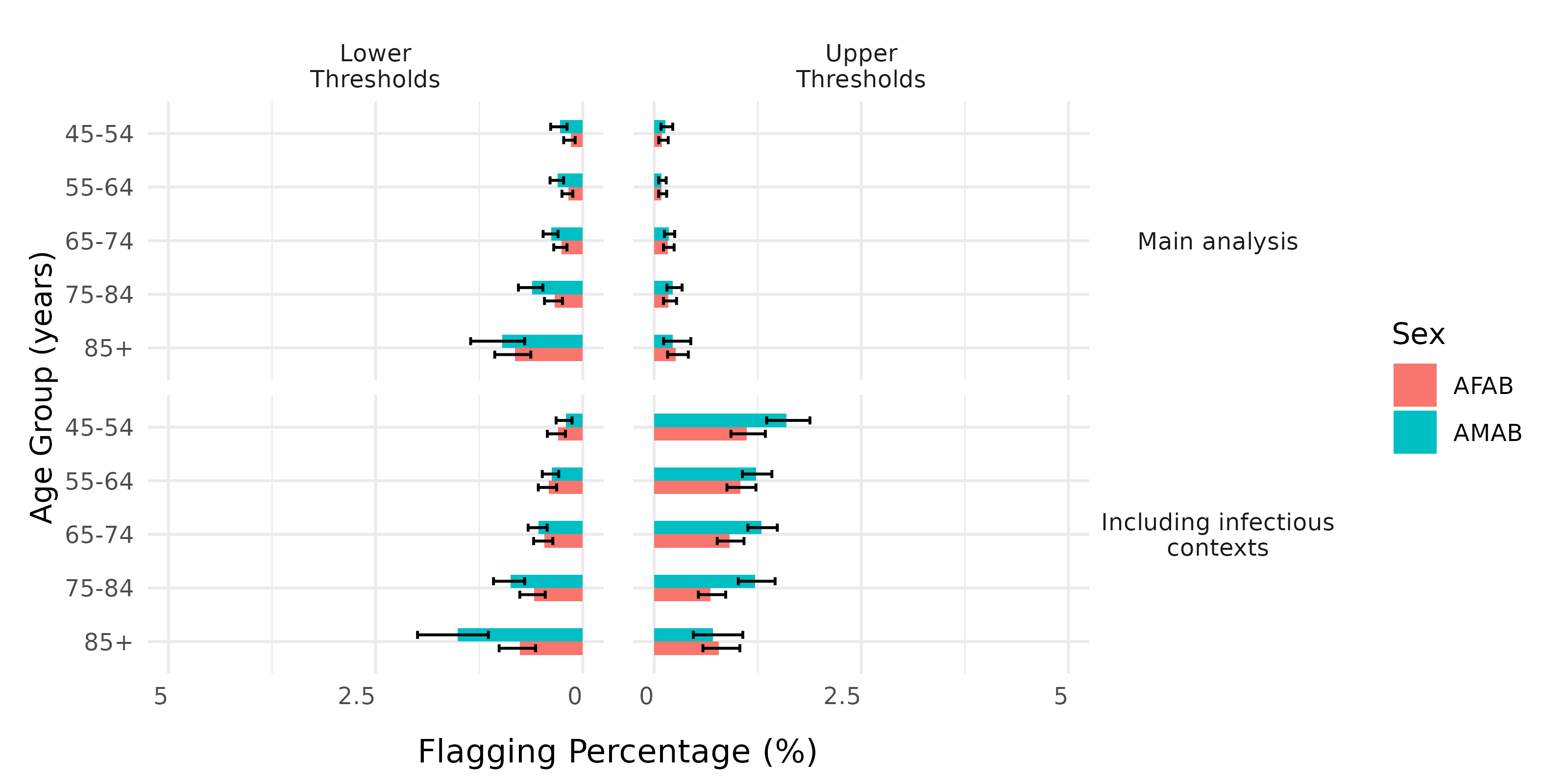

Supplement: S4 Fig — Legend: AFAB: assigned female at birth; AMAB: assigned male at birth. Standard vital signs lower and upper thresholds are 35.0–37.8°C oral equivalent (temperature). 95% Wilson confidence intervals were calculated. (TIFF) [file pone.0349936.s004.tiff]

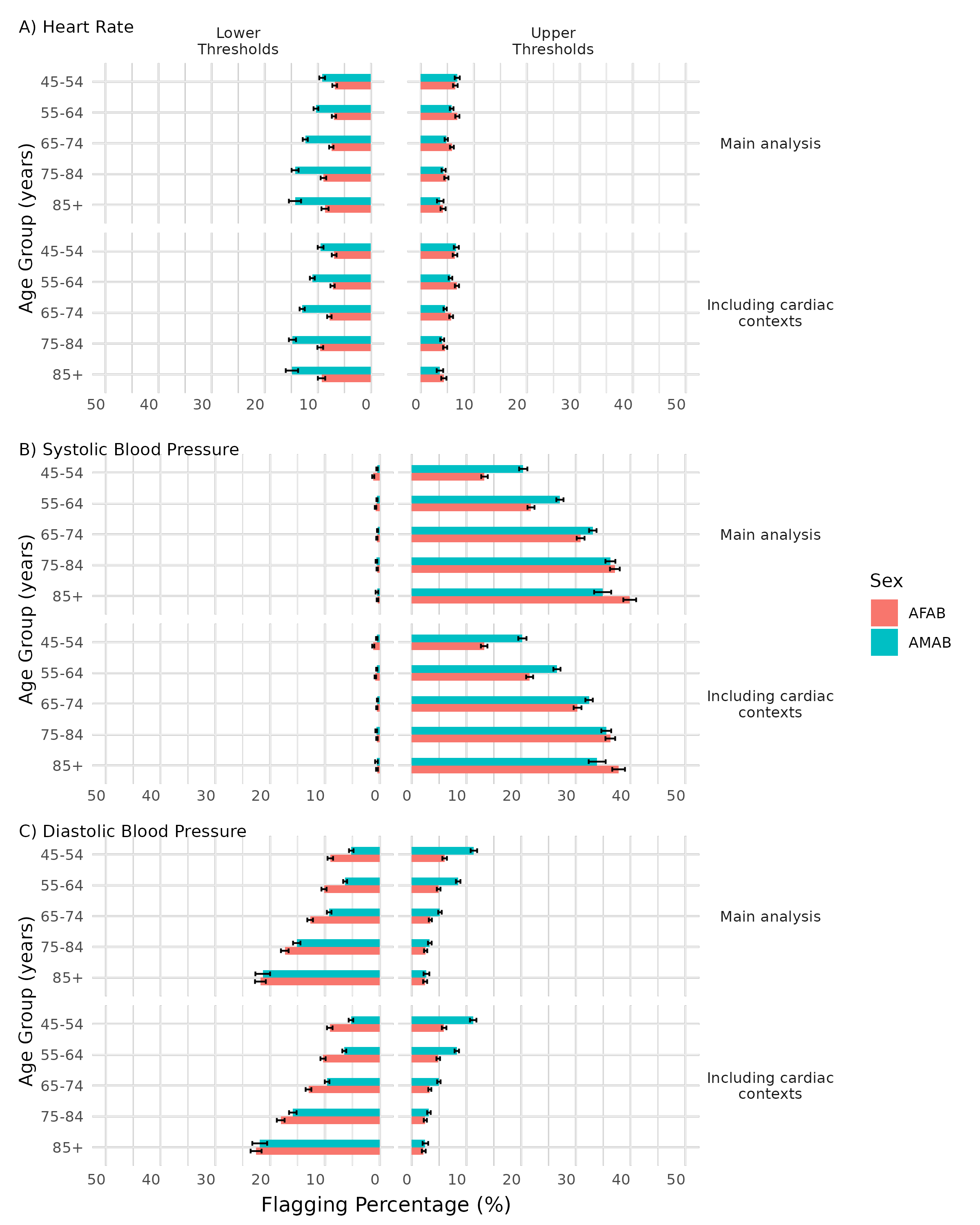

Supplement: S5 Fig — Legend: AFAB: assigned female at birth; AMAB: assigned male at birth. Standard vital signs lower and upper thresholds are 90–140 mmHg (SBP), 60–90 mmHg (DBP) and 60–100 BPM (HR). 95% Wilson confidence intervals were calculated. (TIFF) [file pone.0349936.s005.tiff]
