# Supplementary material for: Older age and sex differences in the proportion of vital signs flagged as abnormal
Source: PLoS One. 2026 May 29;21(5):e0349936. doi: 10.1371/journal.pone.0349936 (PMC13221073; doi:10.1371/journal.pone.0349936)
Supplement: S1 Table — Legend: AFAB: assigned female at birth; AMAB: assigned male at birth; BPM: beats per minute; DBP: diastolic blood pressure; F: female; HR: heart rate; M: male; SBP: systolic blood pressure. The columns representing the upper thresholds are in grey. The centile-based approach was used. 95% Wilson confidence intervals were calculated. (DOCX) [file pone.0349936.s006.docx]

### **S Table 1. Flagging percentages of standard VS thresholds by age group and sex in our study cohort.**

|  | **Age Group (years)** | **HR**  **(BPM)** | | **SBP**  **(mmHg)** | | **DBP**  **(mmHg)** | | **Temperature**  **(°C)** | |
| --- | --- | --- | --- | --- | --- | --- | --- | --- | --- |
|  |  | **100** | **60** | **140** | **90** | **90** | **60** | **37.8** | **35.0** |
| **AFAB** | **45-54** | 6.5 (6.1-7.0) | 6.8 (6.4-7.3) | 13.3 (12.7-13.9) | 1.2 (1.0-1.4) | 6.0 (5.6-6.4) | 9.0 (8.5-9.5) | 0.1 (0.1-0.2) | 0.1 (0.1-0.2) |
|  | **55-64** | 6.9 (6.5-7.3) | 7.0 (6.6-7.4) | 21.8 (21.1-22.4) | 0.8 (0.6-0.9) | 4.9 (4.6-5.2) | 10.2 (9.7-10.7) | 0.1 (0.1-0.2) | 0.2 (0.1-0.3) |
|  | **65-74** | 5.9 (5.5-6.2) | 7.4 (7.1-7.9) | 30.8 (30.1-31.6) | 0.5 (0.4-0.6) | 3.4 (3.1-3.7) | 12.7 (12.2-13.2) | 0.2 (0.1-0.2) | 0.3 (0.2-0.4) |
|  | **75-84** | 4.8 (4.4-5.2) | 8.9 (8.4-9.5) | 37.1 (36.2-38.0) | 0.5 (0.3-0.6) | 2.5 (2.2-2.8) | 17.3 (16.7-18.1) | 0.2 (0.1-0.3) | 0.3 (0.2-0.5) |
|  | **85+** | 4.2 (3.7-4.7) | 8.6 (8.0-9.3) | 39.8 (38.6-41.0) | 0.4 (0.3-0.6) | 2.4 (2.1-2.8) | 21.8 (20.8-22.8) | 0.3 (0.2-0.4) | 0.8 (0.6-1.1) |
| **AMAB** | **45-54** | 6.9 (6.6-7.4) | 9.2 (8.6-9.7) | 20.3 (19.6-21.1) | 0.5 (0.4-0.6) | 11.3 (10.7-11.9) | 5.2 (4.8-5.6) | 0.1 (0.1-0.2) | 0.3 (0.2-0.4) |
|  | **55-64** | 5.8 (5.5-6.2) | 10.3 (9.9-10.8) | 27.1 (26.4-27.7) | 0.5 (0.4-0.6) | 8.5 (8.1-8.9) | 6.3 (6.0-6.7) | 0.1 (0.1-0.1) | 0.3 (0.2-0.4) |
|  | **65-74** | 4.8 (4.5-5.1) | 12.4 (11.9-12.8) | 33.1 (32.4-33.7) | 0.4 (0.3-0.5) | 5.1 (4.8-5.5) | 9.2 (8.8-9.7) | 0.2 (0.1-0.2) | 0.4 (0.3-0.5) |
|  | **75-84** | 4.3 (3.9-4.7) | 14.3 (13.6-14.9) | 36.3 (35.4-37.2) | 0.6 (0.5-0.8) | 3.3 (3.0-3.6) | 15.2 (14.5-15.8) | 0.2 (0.2-0.3) | 0.6 (0.5-0.8) |
|  | **85+** | 3.6 (3.1-4.3) | 14.3 (13.1-15.5) | 34.8 (33.3-36.4) | 0.5 (0.3-0.8) | 2.6 (2.2-3.2) | 21.3 (20.0-22.7) | 0.2 (0.1-0.4) | 1.0 (0.7-1.4) |

Legend: AFAB: assigned female at birth; AMAB: assigned male at birth; BPM: beats per minute; DBP: diastolic blood pressure; HR: heart rate; SBP: systolic blood pressure. The columns representing the upper thresholds are in grey. The centile-based approach was used. 95% Wilson confidence intervals were calculated.
