# Supplementary material for: Older age and sex differences in the proportion of vital signs flagged as abnormal
Source: PLoS One. 2026 May 29;21(5):e0349936. doi: 10.1371/journal.pone.0349936 (PMC13221073; doi:10.1371/journal.pone.0349936)
Supplement: S2 Table — Legend: AFAB: assigned female at birth; AMAB: assigned male at birth; SBP: systolic blood pressure; VS: vital sign. 95% Wilson confidence intervals were calculated. (DOCX) [file pone.0349936.s007.docx]

### **S Table 2. Flagging percentages of standard VS thresholds by age group and sex using 130/80 mmHg SBP/DBP upper thresholds.**

|  | **Age Group (years)** | **SBP (mmHg)** | **DBP (mmHg)** |
| --- | --- | --- | --- |
|  |  | **130** | **80** |
| **AFAB** | **45-54** | 27.0 (26.2-27.8) | 27.0 (26.2-27.82) |
|  | **55-64** | 38.5 (37.7-39.2) | 24.6 (24.0-25.3) |
|  | **65-74** | 49.3 (48.6-50.1) | 18.5 (17.9-19.1) |
|  | **75-84** | 55.4 (54.5-56.4) | 13.7 (13.1-14.4) |
|  | **85+** | 57.5 (56.3-58.7) | 11.4 (10.7-12.2) |
| **AMAB** | **45-54** | 38.6 (37.7-39.5) | 39.4 (38.5-40.3) |
|  | **55-64** | 45.2 (44.4-45.9) | 34.9 (34.2-35.6) |
|  | **65-74** | 51.9 (51.1-52.6) | 25.3 (24.6-25.9) |
|  | **75-84** | 54.5 (53.6-55.5) | 16.9 (16.2-17.6) |
|  | **85+** | 52.2 (50.5-53.8) | 13.2 (12.2-14.4) |

Legend: AFAB: assigned female at birth; AMAB: assigned male at birth; DBP: diastolic blood pressure; SBP: systolic blood pressure; VS: vital sign. 95% Wilson confidence intervals were calculated.
