# Supplementary material for: Older age and sex differences in the proportion of vital signs flagged as abnormal
Source: PLoS One. 2026 May 29;21(5):e0349936. doi: 10.1371/journal.pone.0349936 (PMC13221073; doi:10.1371/journal.pone.0349936)
Supplement: S3 Table — Legend: AFAB: assigned female at birth; AMAB: assigned male at birth; SBP: systolic blood pressure; VS: vital sign. 95% Wilson confidence intervals were calculated. (DOCX) [file pone.0349936.s008.docx]

### **S Table 3. Flagging percentages of standard VS thresholds by age group and setting for 130/80 mmHg SBP/DBP upper thresholds.**

|  | **Age Group (years)** | **SBP (mmHg)** | **DBP (mmHg)** |
| --- | --- | --- | --- |
|  |  | **130** | **80** |
| **Inpatient** | **45-54** | 30.2 (29.5-31.0) | 29.5 (28.8-30.2) |
|  | **55-64** | 38.9 (38.3-39.5) | 25.6 (25.0-26.1) |
|  | **65-74** | 47.4 (46.8-48.0) | 18.5 (18.0-19.0) |
|  | **75-84** | 51.6 (50.9-52.4) | 13.1 (12.6-13.6) |
|  | **85+** | 53.8 (52.7-54.8) | 11.2 (10.6-11.9) |
| **Outpatient** | **45-54** | 37.2 (36.1-38.3) | 40.1 (38.9-41.3) |
|  | **55-64** | 49.1 (48.1-50.1) | 40.3 (39.3-41.3) |
|  | **65-74** | 58.7 (57.7-59.7) | 30.9 (30.0-31.9) |
|  | **75-84** | 64.6 (63.3-65.9) | 21.3 (20.2-22.4) |
|  | **85+** | 63.6 (61.2-65.9) | 15.7 (14.0-17.6) |

Legend: DBP: diastolic blood pressure; SBP: systolic blood pressure; VS: vital sign. 95% Wilson confidence intervals were calculated.
