# Supplementary material for: Older age and sex differences in the proportion of vital signs flagged as abnormal
Source: PLoS One. 2026 May 29;21(5):e0349936. doi: 10.1371/journal.pone.0349936 (PMC13221073; doi:10.1371/journal.pone.0349936)
Supplement: S4 Table — Legend: AFAB: assigned female at birth; AMAB: assigned male at birth. 95% Wilson confidence intervals were calculated. (DOCX) [file pone.0349936.s009.docx]

### **S Table 4. Flagging percentages of standard temperature thresholds by sex when including infectious contexts.**

|  | **Age Group (years)** | **Temperature (°C)** | |
| --- | --- | --- | --- |
|  |  | **37.8** | **35.0** |
| **AFAB** | **45-54** | 0.1 (0.1-0.2) | 0.1 (0.1-0.2) |
|  | **55-64** | 0.1 (0.1-0.2) | 0.2 (0.1-0.3) |
|  | **65-74** | 0.2 (0.1-0.2) | 0.3 (0.2-0.4) |
|  | **75-84** | 0.2 (0.1-0.3) | 0.3 (0.2-0.5) |
|  | **85+** | 0.3 (0.2-0.4) | 0.8 (0.6-1.1) |
| **AMAB** | **45-54** | 0.1 (0.1-0.2) | 0.3 (0.2-0.4) |
|  | **55-64** | 0.1 (0.1-0.1) | 0.3 (0.2-0.4) |
|  | **65-74** | 0.2 (0.1-0.2) | 0.4 (0.3-0.5) |
|  | **75-84** | 0.2 (0.2-0.3) | 0.6 (0.5-0.8) |
|  | **85+** | 0.2 (0.1-0.4) | 1.0 (0.7-1.4) |

Legend: AFAB: assigned female at birth; AMAB: assigned male at birth. 95% Wilson confidence intervals were calculated.
