# Supplementary material for: Older age and sex differences in the proportion of vital signs flagged as abnormal
Source: PLoS One. 2026 May 29;21(5):e0349936. doi: 10.1371/journal.pone.0349936 (PMC13221073; doi:10.1371/journal.pone.0349936)
Supplement: S5 Table — Legend: AFAB: assigned female at birth; AMAB: assigned male at birth; BPM: beats per minute; C: centile; T°: temperature; VS: vital sign. Adapted thresholds with a global flagging percentage of 5% would correspond to the values of the 2.5th and 97.5th centiles (rows in grey). 95% Confidence intervals were bootstrapped with 1000 replicates. (DOCX) [file pone.0349936.s010.docx]

### **S Table 5. Adapted thresholds based on corresponding VS values of centiles when including infectious contexts.**

|  | | **AFAB (age group in years)** | | | | | **AMAB (age group in years)** | | | | |
| --- | --- | --- | --- | --- | --- | --- | --- | --- | --- | --- | --- |
| **VS** | **C** | **45-54** | **55-64** | **65-74** | **75-84** | **85+** | **45-54** | **55-64** | **65-74** | **75-84** | **85+** |
| T°  (°C) | 99 | 37.4 (37.4-37.5) | 37.5 (37.4-37.5) | 37.4 (37.4-37.5) | 37.4 (37.3-37.4) | 37.4 (37.3-37.5) | 37.5 (37.5-37.5) | 37.4 (37.4-37.5) | 37.5 (37.4-37.5) | 37.4 (37.4-37.5) | 37.4 (37.3-37.4) |
|  | 97.5 | 37.3 (37.3-37.3) | 37.3 (37.3-37.3) | 37.3 (37.3-37.3) | 37.2 (37.2-37.2) | 37.2 (37.2-37.2) | 37.3 (37.3-37.3) | 37.3 (37.2-37.3) | 37.3 (37.2-37.3) | 37.2 (37.2-37.3) | 37.2 (37.1-37.2) |
|  | 95 | 37.2 (37.2-37.2) | 37.2 (37.1-37.2) | 37.1 (37.1-37.2) | 37.1 (37.1-37.1) | 37.1 (37.0-37.1) | 37.2 (37.1-37.2) | 37.1 (37.1-37.1) | 37.1 (37.1-37.1) | 37.1 (37.1-37.1) | 37.0 (37.0-37.1) |
|  | 5 | 36.0 (36.0-36.0) | 36.0 (36.0-36.0) | 36.0 (36.0-36.0) | 36.0 (35.9-36.0) | 35.9 (35.8-35.9) | 36.0 (36.0-36.0) | 36.0 (36.0-36.0) | 35.9 (35.9-35.9) | 35.9 (35.8-35.9) | 35.7 (35.6-35.8) |
|  | 2.5 | 35.9 (35.9-35.9) | 35.8 (35.8-35.9) | 35.7 (35.7-35.8) | 35.7 (35.6-35.7) | 35.5 (35.5-35.6) | 35.8 (35.8-35.9) | 35.8 (35.7-35.8) | 35.6 (35.6-35.7) | 35.6 (35.6-35.6) | 35.5 (35.4-35.6) |
|  | 1 | 35.6 (35.6-35.7) | 35.5 (35.5-35.6) | 35.5 (35.5-35.5) | 35.5 (35.4-35.5) | 35.2 (35.0-35.3) | 35.5 (35.5-35.6 | 35.5 (35.5-35.5) | 35.4 (35.4-35.5) | 35.3 (35.2­-35.4) | 35.1 (35.0-35.2) |

Legend: AFAB: assigned female at birth; AMAB: assigned male at birth; BPM: beats per minute; C: centile; T°: temperature; VS: vital sign. Adapted thresholds with a global flagging percentage of 5% would correspond to the values of the 2.5^th^ and 97.5^th^ centiles (rows in grey). 95% Confidence intervals were bootstrapped with 1000 replicates.
