# Supplementary material for: Older age and sex differences in the proportion of vital signs flagged as abnormal
Source: PLoS One. 2026 May 29;21(5):e0349936. doi: 10.1371/journal.pone.0349936 (PMC13221073; doi:10.1371/journal.pone.0349936)
Supplement: S6 Table — Legend: AFAB: assigned female at birth; AMAB: assigned male at birth; BPM: beats per minute; DBP: diastolic blood pressure; HR: heart rate; SBP: systolic blood pressure; VS: vital sign. 95% Wilson confidence intervals were calculated. (DOCX) [file pone.0349936.s011.docx]

**S Table 6. Flagging percentages of standard VS thresholds by sex when including cardiac contexts.**

|  | **Age Group (years)** | **HR (BPM)** | | **SBP (mmHg)** | | **DBP (mmHg)** | |
| --- | --- | --- | --- | --- | --- | --- | --- |
|  |  | **100** | **60** | **140** | **90** | **90** | **60** |
| **AFAB** | **45-54** | 6.5(6.0-6.9) | 6.9 (6.5-7.4) | 13.2 (12.6-13.8) | 1.2 (1.0-1.4) | 5.9 (5.5-6.3) | 9.1 (8.6-9.6) |
|  | **55-64** | 6.8 (6.4-7.2) | 7.2 (6.8-7.6) | 21.5 (20.9-22.2) | 0.8 (0.7-0.9) | 4.8 (4.5-5.2) | 10.4 (9.9-10.8) |
|  | **65-74** | 5.7 (5.4-6.1) | 7.8 (7.4-8.2) | 30.3 (29.6-31.0) | 0.5 (0.4-0.7) | 3.3 (3.0-3.6) | 13.0 (12.5-13.5) |
|  | **75-84** | 4.6 (4.2-5.0) | 9.5 (9.0-10.1) | 36.3 (35.4-37.2) | 0.5 (0.4-0.6) | 2.5 (2.2-2.8) | 18.1 (17.4-18.8) |
|  | **85+** | 4.3 (3.9-4.8) | 9.3 (8.6-10.0) | 37.8 (36.6-38.9) | 0.5 (0.3-0.7) | 2.2 (1.8-2.5) | 22.6 (21.6-23.6) |
| **AMAB** | **45-54** | 6.7 (6.3-7.2) | 9.5 (8.9-10.0) | 20.2 (19.4-21.0) | 0.5 (0.4-0.7) | 11.2 (10.6-11.8) | 5.2 (4.8-5.7) |
|  | **55-64** | 5.6 (5.3-6.0) | 11.0 (10.6-11.5) | 26.5 (25.9-27.2) | 0.5 (0.4-0.7) | 8.2 (7.8-8.6) | 6.5 (6.1-6.8) |
|  | **65-74** | 4.6 (4.3-4.9) | 12.9 (12.5-13.4) | 32.4 (31.7-33.1) | 0.4 (0.3-0.5) | 4.9 (4.6-5.3) | 9.6 (9.2-10.1) |
|  | **75-84** | 4.0 (3.7-4.4) | 14.8 (14.1-15.5) | 35.5 (34.6-36.4) | 0.6 (0.5-0.8) | 3.1 (2.8-3.5) | 15.9 (15.2-16.6) |
|  | **85+** | 3.6 (3.0-4.2) | 14.8 (13.7-16.0) | 33.8 (32.3-35.4) | 0.6 (0.4-0.9) | 2.4 (2.0-3.0) | 21.9 (20.6-23.3) |

Legend: AFAB: assigned female at birth; AMAB: assigned male at birth; BPM: beats per minute; DBP: diastolic blood pressure; HR: heart rate; SBP: systolic blood pressure; VS: vital sign. 95% Wilson confidence intervals were calculated.
