# Supplementary material for: Older age and sex differences in the proportion of vital signs flagged as abnormal
Source: PLoS One. 2026 May 29;21(5):e0349936. doi: 10.1371/journal.pone.0349936 (PMC13221073; doi:10.1371/journal.pone.0349936)
Supplement: S7 Table — Legend: AFAB: assigned female at birth; AMAB: assigned male at birth; BPM: beats per minute; C: centile; DBP: diastolic blood pressure; HR: heart rate; SBP: systolic blood pressure; VS: vital sign. Adapted thresholds with a global flagging percentage of 5% would correspond to the values of the 2.5th and 97.5th centiles (rows in grey). 95% Confidence intervals were bootstrapped with 1000 replicates. (DOCX) [file pone.0349936.s012.docx]

**S Table 7. Adapted thresholds based on corresponding VS values of centiles when including cardiac contexts.**

|  | | **AFAB (age group in years)** | | | | | **AMAB (age group in years)** | | | | |
| --- | --- | --- | --- | --- | --- | --- | --- | --- | --- | --- | --- |
| **VS** | **C** | **45-54** | **55-64** | **65-74** | **75-84** | **85+** | **45-54** | **55-64** | **65-74** | **75-84** | **85+** |
| HR  (BPM) | 99 | 114 (113-116) | 115 (114-116) | 113 (112-114) | 112 (110-114) | 112 (110-114) | 115 (114-117) | 113 (112-114) | 111 (110-112) | 111 110-112) | 112 (109-114) |
|  | 97.5 | 108 (107-109) | 109 (108-109) | 107 (106-108) | 105 (104-106) | 105 (104-106) | 108 (108-110) | 107 (106-107) | 105 (105-106) | 105 (104-106) | 103 (102-105) |
|  | 95 | 103 (102-103) | 103 (103-104) | 102 (101-102) | 100 (100-100) | 100 (99-100) | 103 (102-104) | 102 (101-102) | 100 (100-100) | 99 (99-100) | 98 (97-99) |
|  | 5 | 59 (59-59) | 59 (59-59) | 59 (58-59) | 58 (58-58) | 59 (58-59) | 57 (57-58) | 57 (56-57) | 55 (55-56) | 55 (55-56) | 56 (55-56) |
|  | 2.5 | 56 (55-56) | 56 (55-56) | 56 (55-56) | 55 (55-55) | 56 (55-56) | 54 (54-55) | 54 (53-54) | 52 (52-53) | 52 (52-53) | 52 (51-53) |
|  | 1 | 52 (52-53) | 52 (52-53) | 52 (52-53) | 52 (51-52) | 53 (52-54) | 51 (51-52) | 51 (50-51) | 49 (49-50) | 49 (49-50) | 50 (48-50) |
| SBP  (mm Hg) | 99 | 166 (165-168) | 173 (171-175) | 178 (177-180) | 184 (182-185) | 186 (184-188) | 172 (169-172) | 175 (173-176) | 178 (177-180) | 181 (179-182) | 179 (177-182) |
|  | 97.5 | 157 (156-159) | 164 (164-165) | 170 (170-171) | 176 (175-177) | 177 (175-179) | 162 (161-163) | 166 (166-167) | 170 (169-170) | 173 (172-174) | 174 (172-175) |
|  | 95 | 151 (150-151) | 158 (157-158) | 164 (164-165) | 169 (168-170) | 170 (169-171) | 156 (155-157) | 160 (159-160) | 163 (163-164) | 167 (166-167) | 168 (166-169) |
|  | 5 | 97 (97-98) | 100 99-100) | 102 (102-103) | 103 (103-104) | 103 (102-104) | 101 (101-102) | 102 (102-102) | 103 (103-103) | 102 (101-102) | 102 (101-102) |
|  | 2.5 | 94 (93-94) | 96 (95-96) | 98 (97-99) | 99 (99-100) | 99 (98-100) | 98 (97-98) | 98 (98-99) | 99 (98-100) | 97 (97-98) | 97 (96-99) |
|  | 1 | 90 (90-91) | 91 (91-92) | 93 (93-94) | 94 (93-95) | 94 (93-95) | 94 (92-94) | 94 (93-94) | 94 (93-94) | 93 (92-94) | 93 (91-94) |
| DBP  (mm Hg) | 99 | 99 (99-100) | 98 (97-99) | 96 (96-97) | 95 (94-96) | 94 (93-95) | 105 (104-106) | 100 (100-102) | 98 (97-98) | 96 (95-97) | 96 (94-98) |
|  | 97.5 | 95 (94-95) | 94 (93-94) | 92 91-92) | 90 (90-91) | 90 (89-91) | 99 (99-100) | 97 (96-97) | 94 (93-94) | 92 (91-92) | 90 (89-92) |
|  | 95 | 92 (91-92) | 90 (90-91) | 88 (88-89) | 87 (86-87) | 85 (85-86) | 96 (95-96) | 93 (93-94) | 90 (90-91) | 88 (88-88) | 86 (85-87) |
|  | 5 | 58 (57-58) | 57 (57-58) | 56 (55-56) | 53 (53-54) | 52 (52-53) | 60 (60-61) | 60 (60-60) | 57 (57-58) | 54 (54-55) | 52 (52-53) |
|  | 2.5 | 54 (54-55) | 54 (54-54) | 52 (52-53) | 50 (50-51) | 50 (49-50) | 58 (57-58) | 56 (56-57) | 54 (54-55) | 51 (50-52) | 50 (49-50) |
|  | 1 | 51 (50-52) | 50 (50-50) | 49 (49-50) | 47 (46-48) | 46 (44-48) | 54 (53-54) | 52 (52-53) | 50 (50-51) | 48 (47-48) | 47 (45-48) |

Legend: AFAB: assigned female at birth; AMAB: assigned male at birth; BPM: beats per minute; C: centile; DBP: diastolic blood pressure; HR: heart rate; SBP: systolic blood pressure; VS: vital sign. Adapted thresholds with a global flagging percentage of 5% would correspond to the values of the 2.5^th^ and 97.5^th^ centiles (rows in grey). 95% Confidence intervals were bootstrapped with 1000 replicates.
