# Supplementary material for: Older age and sex differences in the proportion of vital signs flagged as abnormal
Source: PLoS One. 2026 May 29;21(5):e0349936. doi: 10.1371/journal.pone.0349936 (PMC13221073; doi:10.1371/journal.pone.0349936)
Supplement: S1 File — (DOCX) [file pone.0349936.s013.docx]

# **Section 1. eMethods**

## **1.1 Groups for the first objective analysis and subgroup analysis**

Groups for the first objective analysis were made for each VS (HR, SBP, DBP, and temperature), age group (45-54y, 55-64y, 65-74y, 75-84y, and 85y+), and sex assigned at birth (female, or male). For the subgroup analysis according to setting, the groups were made for each VS (HR, SBP, DBP, and temperature), age group (45-54y, 55-64y, 65-74y, 75-84y, and 85y+), and setting (inpatient, or outpatient).
