# Supplementary material for: Older age and sex differences in the proportion of vital signs flagged as abnormal
Source: PLoS One. 2026 May 29;21(5):e0349936. doi: 10.1371/journal.pone.0349936 (PMC13221073; doi:10.1371/journal.pone.0349936)
Supplement: S2 File — (DOCX) [file pone.0349936.s014.docx]

# **Section 2. eResults**

## **2.1 Building of the cohort**

Of the 9,653,672 patient visits available in the CITADEL database, there were 257,844 patient visits that met the inclusion and exclusion criteria. Inclusion criteria were visits with at least one vital sign, available data on sex and date of vital sign measurement, last vital signs set before hospital discharge for inpatients, temperature routes (oral, rectal, or unspecified), and departments other than those with risk of being in the acute phase of an illness (intensive care, palliative care, and emergency department), or with very limited data. Of the remaining 257,844 patients visits, 190,867 were inpatient visits and 66,977 were outpatient visits. The exclusion criteria were then applied: exclusion of VS values considered implausible (n = 35), death during hospitalization (n = 4,737), and the random selection of one VS set per unique patient if more than one hospitalization or outpatient visit (n = 129,652). The final cohort included 123,420 unique patients with one VS set that was complete or partial. Analysis for each individual vital sign did not include the 123,420 unique patients, but each included patient contributed to at least one analysis. Of the 123,420 patients, there was data on HR for 111,371, on SBP for 112,162, on DBP for 112,162, and on temperature for 103,395 patients. When the temperature routes were specified, the sites were axillar (n = 265; 1%), central (n = 11; <0.1%), cutaneous (n = 4,251; 16%), oral (n = 20,824; 80%), rectal (n = 489; 1.9%), and not available (n = 69; 0.3%). The temperature route was not specified for 97,511 measures in the cohort. These measures were considered as oral site since the distribution was similar and it is common in clinical practice not to specify the site in case of routine oral temperature. The medical department specialties comprised: cardiology, clinical immunology/allergy endocrinology, gastroenterology, general practice, geriatric medicine, hematology-oncology, hepatology, infectious disease, internal medicine, nephrology, neurology, respirology, and rheumatology. The surgical department specialties comprised: cardiovascular and thoracic surgery, general surgery, maxillofacial surgery, neurosurgery, obstetrics and gynecology, ophthalmology, orthopedic surgery, otolaryngology, plastic surgery, preoperative clinic, transplant, urology, and vascular surgery. The other department specialties comprised: nursing, psychiatry, radiation oncology, and radiology.

## **2.2 Sensitivity analyses of centile-based thresholds based on statistical method.**

There were warnings for non-convergence of the GAMLSS model. The subgroups with non-convergence were: temperature in AFAB of the 65-74 years and 75-84 years groups, and temperature in AMAB of the 65-74 years and 75-84 years groups. Results are displayed in Figs 3A and 3B.
